# A systematic review of the impact of volume of surgery and specialization in Norwood procedure

**DOI:** 10.1186/1471-2431-14-198

**Published:** 2014-08-06

**Authors:** Dawid Pieper, Tim Mathes, Boulos Asfour

**Affiliations:** 1 Institute for Research in Operative Medicine, Witten/Herdecke University, Ostmerheimer Str. 200, Building 38, Cologne D- 51109, Germany; 2 German Pediatric Heart Centre, Asklepios Clinic Sankt Augustin, Sankt Augustin, Germany

**Keywords:** Norwood procedures, Hypoplastic left heart syndrome, Outcome assessment (Health care), Mortality, Review

## Abstract

**Background:**

The volume-outcome relationship is supposed to be stronger in high risk, low volume procedures. The aim of this systematic review is to examine the available literature on the effects of hospital and surgeon volume, specialization and regionalization on the outcomes of the Norwood procedure.

**Methods:**

A systematic literature search was performed in Medline, Embase, and the Cochrane Library. On the basis of titles and abstracts, articles of comparative studies were obtained in full-text in case of potential relevance and assessed for eligibility according to predefined inclusion criteria. All relevant data on study design, patient characteristics, hospital volume, surgeon volume and other institutional characteristics, as well as results were extracted in standardized tables. Study selection, data extraction and critical appraisal were carried out independently by two reviewers.

**Results:**

We included 10 studies. All but one study had an observational design. The number of analyzed patients varied from 75 to 2555. Overall, the study quality was moderate with a huge number of items with an unclear risk of bias. All studies investigating hospital volume indicated a hospital volume-outcome relationship, most of them even having significant results. The results were very heterogeneous for surgeon volume.

**Conclusions:**

The volume-outcome relationship in the Norwood procedure can be supported. However, the magnitude of the volume effect is difficult to assess.

## Background

Previous systematic reviews (SR) have shown the presence of a significant volume-outcome relationship in surgery [1-6]. This relationship is supposed to be stronger in high risk, low volume procedures [7-10]. Two hypotheses exist for this relationship. On the one hand, a higher caseload and experience result in more effective skills (“practice makes perfect”). On the other hand, providers with better outcomes might receive more referrals increasing their volume (“selective referral”) [11,12].

Among the termination of pregnancy, compassionate care and heart transplantation, surgical palliation is the fourth treatment option for hypoplastic left heart syndrome (HLHS). A prevalence of 0.016 to 0.025% has been reported for hypoplastic left heart syndrome in neonates [13,14]. Infants suffering from hypoplastic left heart syndrome may undergo a three-stage reconstruction. The Norwood procedure is the first (stage 1 palliation) operation of a series of three operations. Surgical details on the surgical technique of the Norwood procedure can be found elsewhere [15,16]. After the Norwood procedure children will generally undergo the Glenn (stage 2 palliation at 3 to 6 months of age) and Fontan procedure (stage 3 palliation at 18 to 48 months of age) [17].

The Norwood procedure is associated with high mortality rates, varying between 10 and 35% [18-24]. It has been debated whether mortality rates differ by provider volume or specialization [25-28]. The introduction of minimal volume standards or other interventions leading to centralized care might be of high interest for decision-makers.

Individual studies investigating quality differences between pediatric cardiac surgical centers are known to be often underpowered [29]. To the best of our knowledge, no SR on the volume-outcome relationship in the Norwood procedure exists. The aim of this systematic review is to examine the available literature on the effects of hospital and surgeon volume, specialization and regionalization on the outcomes of the Norwood procedure.

## Methods

We performed a systematic literature search to identify all relevant publications on the relationship between provider volume or specialization and clinical outcomes. Medline (via PubMed), Embase (via Embase) and all databases of the Cochrane library were searched from inception to March 2013 (see Additional file 1 for search strategies). Reference lists of relevant articles were inspected to identify additional articles that could have been missed by our search strategy. No language restrictions were applied.

To be considered in this systematic review the following inclusion criteria were applied to each publication: the subject of the study was the Norwood procedure; the study had a comparative design; patient outcomes (e.g. mortality, morbidity) were studied; volume (if applicable) was defined as a distinct number (e.g. continuous variable) or a cut-off value, or specialized hospitals/units were analyzed; the study did not describe a single hospital or surgeon. All titles and abstracts were screened independently by two members of the research team and the full texts of potentially eligible articles were then obtained and further assessed for eligibility according to the review inclusion criteria. Any disagreements were resolved by discussion.

Data were extracted by one reviewer into structured summary tables and checked for accuracy by a second reviewer. Any disagreements were resolved through discussion until consensus was reached. For each publication, we extracted data on patient characteristics; setting; data source(s); study design and methodology; model adjustments; independent variable in terms of provider volume, specification or regionalization; and results.

The methodological quality of the eligible studies was assessed independently by two reviewers. Any disagreements were resolved by discussion. We modified a tool which is based on the Newcastle-Ottawa-Scale [30] that was recently used in a Cochrane review investigating the volume-outcome relationship in colorectal cancer [31]. As many of the identified studies were expected to be registry-based, we made some minor changes to the tool. We believe that the last two questions dealing with incomplete data and missing data cannot be applied to registry-based studies. For example, registries might only incorporate data on cases with complete data. Under these circumstances a question on incomplete or missing data would be pointless. Therefore, we replaced these two questions for all registry-based studies and evaluated the “quality of registry data” and the “selection of patients” instead. Both questions were previously used for a similar question related to the volume-outcome relationship in registry-based studies [32]. For all other studies we used the original assessment tool of the Cochrane review by Archampong et al. [31]. In contrast to clinical trials, all registry-based studies were assessed to have a high risk of bias in the study design item in the review. We omitted this item as it seems inappropriate to assess retrospective study designs per se to be at high risk of bias with respect to our study objective. Information in registry-based studies is obtained prospectively. There is no obvious reason why registry-based studies should be at high risk of bias due to their design. Our modified assessment tool can be found in the Additional file 2.

Because the identified studies were expected to be clinically and methodologically diverse (for example, different volume definitions), we decided a priori not to statistically combine results.

## Results

### Study selection and characteristics

**Figure 1:**
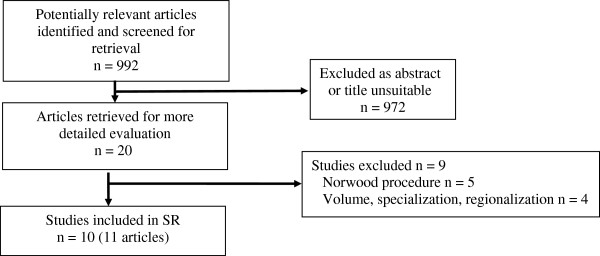
Flow chart.

The search strategy generated 992 hits, of which 10 studies [33,23,42] (11 publications) met our inclusion criteria (see Figure 1). Additional file 3 lists the excluded studies, along with the reasons for exclusion.

One study was described in two publications [37,38]. All but one study [41] had an observational design. Eight studies were based on registry data [33,42,40,38,23], whereas two were based on clinical trial data [39,41]. We included two studies that included a subgroup analysis for the Norwood procedure [34,23]. All studies were performed in the US, one study additionally included patients from Canada [39]. The number of analyzed patients varied from 75 to 2555. The observation periods differed widely across studies, as well (1 to 19 years). The characteristics of the studies can be found in Table 1.

**Table 1:** Study characteristics

***Author (pub year)***	***Study type***	***Region/country***	***Data source***	***Period***	***Patient characteristics***	***Case definition (ICD-9-CM)***
Chang 2002 [34] (subgroup analysis)	Registry-based	US	NIS	1988-1997	Age ≤30 days at admission	746.7
Gutgesell [35] 2002	Registry-based	US	UHC	1990-1999	Age ≤30 days at admission	746.7 + 39.61/34.42/37.4/38.34 to 38.85/39.56/39.0
Checchia 2005 [42]	Registry-based	US	PHIS	1998-2001	Age ≤30 days at admission	746.7 + 39.0
Berry 2006 [33]	Registry-based	US	KID 1997 and 2000	1997 and 2000	*Teaching vs. non-teaching:*	746.5, 746.7, 747.10 + 39.0, 747.22
					Age at admission in days, median (IQR): 1(0–6)/3(1–6)	
					Male (%) 77/60	
					White (%) 64/62	
					Medicaid (%) 32/49	
					Noncardiac structural anomaly (%) 5/5	
					Prematurity/low birth weight (%) 7/5	
					Aortic atresia (%) 3/2	
					Chromosomal anomaly (%) 3/0	
Hirsch 2008 [36]	Registry-based	US	KID 2003	2003	Male 63.1%	746.7 + 39.0
					White 39.6%	
					Non-white 33.5%	
					Race unknown 26.9%	
Welke 2009 [23] (subgroup analysis)	Registry-based	US	STS-CHSDB	2002-2006	NR	NR
Karamlou 2010 [39]	Clinical study	US + Canada	CHSS studies	1994-2000	NR	NR
McHugh 2010 [40]	Registry-based	US	UHC	1998-2007	NR	746.7 + 34.42/39.0/35.92/37.4/(38.34 to 38.85 + 39.61)
Hornik/Pasquali 2012 [37,38]	Registry-based	US	STS-CHSDB	2000-2009	Mean age (days) 6 (IQR 4–9)	
					Mean weight (kg) 3.18 (IQR 2.80-3.50)	
					Weight <2.5 kg 9.7%	
					Male 58.2%	
					Noncardiac/genetic abnormality 19.9%	
					Dominant ventricle: right 89.6%, left 8.0%,	
					TAPVR 1.3%	
					Mechanical ventilator support 39.9%	
					Mechanical circulatory support 0.8%	
					Shock 6.7%	
					Arrhythmia 2.6%	
					Neurologic deficit 1.3%	
					Complete atrioventricular block 0.2%	
					LOS >7 days 20.8%	
Tabbutt 2012 [41]	Clinical study	US	Pediatric Heart Network SVR trial	05/2005-07/2008	NR	NR

NR not reported, KID Kids’ inpatient database, NIS Nationwide Inpatient Sample, PHIS Pediatric Health Information System, SD standard deviation, UHC University HealthSystem Consortium, STS-CHSDB Society of Thoracic Surgeons Congenital Heart Surgery Database, IQR interquartile range, TAPVR total anomalous pulmonary venous return, CHSS Congenital heart surgeons society, SVR Single Ventricle Reconstruction, y year.

Ten studies investigated the effect of hospital volume [33,42,39,38,41,23]. In four of these studies the authors also investigated surgeon volume [42,37,39,38,41]. In addition to hospital volume, two studies analyzed the data by hospital type [33,36]. Most studies employed regression models for analysis. The results of the studies are shown in Table 2.

**Table 2:** Study results

***Author (pub year)***	***N analysed***	***Hospital type***	***Surgeon volume***		***Hospital volume***		***Model (adjustments)***
Chang 2002 [34]	78 (1988–1992), 268 (1993–1997)	NI	NI			Hospital mortality	No model (volume treated as continuous variable; correlation coefficients)
					1988-1992	r = -0.20 (p < 0.01)	
					1993-1997	r = -0.31 (p < 0.01)	
Gutgesell 2002 [35]	1203	NI		NI		Mortality	No model
					Low ≤ 50	50%	
					High >50	40%	
Checchia 2005 [42]	801	NI		Survival		Survival	No model
			Low ≤4	49%	Low <16	48%	
			High >4	69%	Medium 16-30	62%	
					High >30	71%	
						p = 0.08	
						Increase by 4% (95% CI 1%-7%) for every 10 additional procedures performed	Linear regression
				Trend for mortality p = 0.13		Trend for mortality p = 0.02	Volume treated as continuous variable
				Association of risk-unadjusted mortality		Association of risk-unadjusted mortality	
				r^2^ = NR, p = 0.312		r^2^ = 0.18, p = 0.02	
						Mean LOS survivors (SD)	
					Low <16	36.5 ± 32.4	
					Medium 16-30	28.7 ± 8.4	
					High >30	29.4 ± 5.7	
						p > 0.05	
						Mean TTD (SD)	
					Low <16	19.6 ± 33.2	
					Medium 16-30	12.2 ± 9.7	
					High >30	20.2 ± 9.6	
						p > 0.05	
						Median TTD (range)	
					Low <16	19.2 (1–104)	
					Medium 16-30	5.4 (1–13)	
					High >30	7.8 (1–27)	
						p > 0.05	
Berry 2006 [33]	754 (1997), 880 (2000)	Non-teaching vs. teaching	Hospital mortality	NI		Hospital mortality	Logistic regression (teaching status, hospital volume, noncardiac structural anomaly, prematurity, low birth weight, aortic atresia, chromosomal anomaly)
		OR 2.6 (1.3 - 5.3)			Low	OR 3.1 (1.1 – 8.3)	
					Mid-low	OR 2.0 (0.7 – 5.7)	
					Mid-high	OR 1.0 (0.5 – 1.8)	
					High	reference	
					(Categories were determined from quartiles)		
Hirsch 2008 [36]	624		Hospital mortality			Mortality	
		Urban teaching	24.4%			Inverse association p = 0.0001	Logistic regression (volume treated as continuous variable)
		Urban non-teach.	32.2%				
		Rural	34.0%				
		Unknown	26.6%				
Welke 2009 [23]	1154	NI	NI		Mortality		Logistic regression (age, age-for-weight-and-sex z score, interaction between age and age-for-weight-and-sex z score, preoperative stay for more than 2 days; number of prior operations (0, 1, ≤2); renal failure or dialysis, acidosis, circulatory support or shock; preoperative ventilator support or tracheostomy; asplenia, polysplenia, or a22q11 deletion; DiGeorge syndrome; Down syndrome; procedure or procedure group; operation date
				Low <150	OR 2.91 (1.98-4.28)		
				Medium 150-249	OR 1.59 (1.09-2.32)		
				High 250-349	OR 1.43 (1.06-1.95)		
				Very high ≥350	Reference		
					Volume categories for pediatric cardiac surgery	p = 0.002 when hospital volume analysed as continuous variable (test of no volume mortality relationship)	
Karamlou 2010 [39]	710	NI		Mortality		Mortality	Hazard regression (Birth weight, age at operation, circulatory arrest time, ascending aortic dimension, reimplantation of the ascending aorta, shunt origin from the aorta)
				Increased cases per year (per case): -0.004 ± 0.007 (p = 0.49) [parameter estimate ± SE]		Increased cases per year (per case): -0.005 ± 0.01 (p = 0.38) [parameter estimate ± SE]	
McHugh 2010 [40]	1949	NI	NI			Hospital mortality	Logistic regression (?)
				Low <20		OR 2.49 (1.51-4.07)	
				Medium 20-64		OR 1.75 (1.23-2.49)	
				High >64		Reference	
Hornik/Pasquali 2012 [37,38]	2555	NI		Hospital mortality		Hospital mortality	Logistic regression (all patient characteristics, hospital volume/surgeon volume)
			Low ≤5	OR 1.47 (1.01-2.15	Low ≤10	OR 1.37 (0.92-2.05)	
			Medium 6-10	OR 1.26 (0.88-1.78)	Medium 11-20	OR 1.20 (0.80-1.82)	
			High >10	Reference	High >20	Reference	
						OR 1.17 (1.01-1.35) for a twofold decrease in hospital volume	Volume treated as continuous variable
Tabbutt 2012 [41]	549	NI		Renal failure		Renal failure	Logistic regression (anomalous pulmonary venous return, preoperative intubation, heart block, open sternum, volume)
			Low ≤5	OR 0.31 (0.09-1.09)	Low ≤15	OR 1.55 (0.53-4.58)	
			Medium 6 to 10	OR 0.90 (0.28-2.91)	Medium 16 to 20	OR 0.44 (0.14-1.45)	
			High 11 to 15	OR 0.20 (0.06-0.61)	High 21 to 30	OR 0.32 (0.11-0.91)	
			Very high >15	Reference	Very high >30	Reference	
				Log time to first extubation in days		Log time to first extubation in days	Linear regression (gestational age, left atrial decompression, TR preoperatively, duration of regional cerebral perfusion, ECMO, open sternum, duration of open sternum, operations after Norwood procedure, volume)
			Low ≤5	0.54	Low ≤15	-0.06	
			Medium 6 to 10	0.54	Medium 16 to 20	0.31	
			High 11 to 15	0.40	High 21 to 30	0.21	
			Very high >15	Reference	Very high >30	Reference	
				Log length of ventilation in days		Log length of ventilation in days	Linear regression (gestational age, genetic abnormality, preoperative intubation, left atrial decompression, preoperative shock, TR preoperatively, age, open sternum, operations after Norwood procedure, volume)
			Low ≤5	0.33	Low ≤15	0.004	
			Medium 6 to 10	0.27	Medium 16 to 20	0.26	
			High 11 to 15	0.21	High 21 to 30	0.12	
			Very high >15	Reference	Very high >30	Referene	
						Log time hospital LOS in days	Linear regression (birth weight, genetic abnormality, preoperative intubation for shock, TR preoperative, duration of DHCA, operations after Norwood procedure, volume)
					Low ≤15	0.16	
					Medium 16 to 20	0.34	
					High 21 to 30	-0.03	
					Very high >30	reference	
						Sepsis	Logistic regression (gestational age, AS/MS/VD, duration of DHCA, open sternum duration, volume)
					Low ≤15	OR 2.28 (1.17-4.47)	
					Medium 16 to 20	OR 0.94 (0.40-2.19)	
					High 21 to 30	OR 0.64 (0.33-1.26)	
					Very high >30	reference	

AS/MS/VSD aortic stenosis, mitral stenosis, ventricular septal defect, DHCA deep hypothermic circulatory arrest, ECMO extracorporeal membrane oxygenation, TR tricuspid regurgitation, LOS length of stay, SE standard error, TTD time to death, NI not investigated, NR not reported, OR odds ratio, SD standard deviation.

### Study quality

Table 3 summarizes the results of the quality assessment. More than half of the items were judged to have an unclear risk of bias. Only one item in one study had a high risk of bias. Addressing incomplete data or quality of registry data was the major flaw. For this item all studies had an unclear risk of bias. Many studies had also an unclear risk of bias with respect to the representativeness of the study cohort and the comparability of the intervention and control group. All but one study had a low risk of bias with respect to the assessed outcomes.

**Table 3:** Study quality

***Study***	***Representativeness of study cohort***	***Ascertainment of intervention***	***Comparability of intervention and comparison/control group***	***Assessment of outcomes***	***Addressing incomplete data/quality of registry data***	***Missing data on primary interventions and outcomes/selection of patients***
*Register based studies*						
Chang 2002 [34]	**?**	**?**	**?**	**+**	**?**	**+**
Gutgesell 2002 [35]	**?**	**?**	**?**	**+**	**?**	**+**
Checchia 2005 [42]	**?**	**+**	**?**	**+**	**?**	**-**
Berry 2006 [33]	**+**	**+**	**+**	**+**	**?**	**+**
Hirsch 2008 [36]	**+**	**+**	**?**	**+**	**?**	**+**
Welke 2009 [23]	**?**	**+**	**?**	**+**	**?**	**?**
McHugh 2010 [40]	**?**	**?**	**?**	**+**	**?**	**+**
Hornik/Pasquali 2012 [37,38]	**+**	**?**	**+**	**+**	**?**	**?**
*clinical studies*						
Karamlou 2010 [39]	**?**	**+**	**?**	**+**	**?**	**?**
Tabbutt 2012 [41]	**?**	**+**	**?**	**?**	**?**	**?**

+ Low risk of bias, - High risk of bias, ? Unclear risk of bias.

### Hospital type

Berry et al. [33] found non-teaching hospitals to have a significantly higher hospital mortality (OR 2.6, 95% CI 1.3 - 5.3) when compared to teaching hospitals in a multivariate analysis based on the 1997 Kids Inpatient Database (KID). According to the authors’ analyses on the 2000 dataset (not shown) resulted in the same findings.

Hirsch et al. [36] analyzed 60 hospitals based on the Kids Inpatient Database 2003 and found the hospital mortality to be lowest in urban teaching hospitals (24.4%). This is more than 7 and 9% points lower than for urban non-teaching and rural hospitals, respectively. For more than one in four hospitals (26.6%) the type was unknown. However, these results are based on 624 Norwood procedures, 551 (88.3%) of them were performed in urban teaching hospitals.

### Surgeon volume

Checchia et al. [42] analyzed the Pediatric Health Information System (PHIS) from 1998 to 2001. Surgeons with more than 4 Norwood procedures were defined as high volume and compared to their colleagues. Survival was higher in high volume surgeons (69% vs. 49%). Further analyses showed also a trend for mortality (treating surgeon volume as a continuous variable) and an association between the risk-unadjusted mortality and surgeon volume. However, all results did not reach statistical significance.

The Society of Thoracic Surgeons Congenital Heart Surgery Database (STS-CHSDB) was utilized to investigate the surgeon volume during a ten-year period [37,38]. Low volume surgeons (≤5 procedures) had higher mortality rates when compared to high volume surgeons with more than 10 procedures (OR 1.47, 95% CI 1.01 – 2.15). Medium volume (6–10 procedures) surgeons had also higher mortality rates, but this finding was statistically not significant (OR 1.26, 95% CI 0.88-1.78).

Morbidity outcomes were investigated in the Pediatric Heart Network Single Ventricle Reconstruction (SVR) trial, running from May 2005 to July 2008 [41]. Surgeon volume was classified in four categories in intervals of five procedures. Results showed no clear volume-outcome relationship for renal failure. The chance for suffering from renal failure was highest in the highest surgeon volume group. However, findings supported a surgeon volume-outcome relationship for the time to first extubation and for the length of ventilation.

Karamlou et al. [39] did not define volume categories but treated surgeon volume solely as a continuous variable. The results of 56 surgeons who performed 710 procedures from 1994 to 2000 revealed no statistically significant relationship between surgeon volume and mortality based on the analysis of an increase of one additional case per year (p = 0.49).

### Hospital volume

Hospital mortality was associated with hospital volume based on an analysis of the Kids Inpatient Database 1997 [33]. Statistical significance was only reached when low volume hospitals were compared with high volume hospitals (OR 3.1, 95% CI 1.1 – 8.3). Mid-low volume hospitals had a higher chance although statistical significance was not reached (OR 2.0, 95% CI 0.7 – 5.7), whereas mid-high volume hospitals had the same chance as high volume hospitals (OR 1.0, 95% CI 0.5 – 1.8). As already stated above, according to the authors, analyses on the 2000 dataset resulted in the same findings (not shown). Hirsch et al. [36] analyzed the Kids Inpatient Database 2003 dataset and found a highly significant hospital volume-outcome relationship based on data from 60 hospitals (p < 0.0001). A former study supports this inverse association between hospital mortality and hospital volume [34]. The correlation coefficients were r = -0.20 (p < 0.01) for the period 1988–1992 and even r = -0.31 (p < 0.01) for the next period (1993–1997).

The PHIS (data 1998 – 2001) was utilized to investigate the hospital volume-outcome relationship with three categories in intervals of 15 procedures [42]. Although there was a tendency for higher survival in high volume hospitals (high vs. medium vs. low, 71% vs. 62% vs. 48%) this turned out not to be significant (p = 0.08). Further analyses showed also a relationship for mortality (treating hospital volume as a continuous variable (p = 0.02) and an association between risk-unadjusted mortality (r^2^ = 0.18) and hospital volume (p = 0.02). Furthermore, the survival improved by 4% (95% CI 1-7%) for every 10 additional procedures performed. The hospital volume had no significant influence on the length of stay and the time to death (analyzed as mean and median).

McHugh et al. [40] analyzed data on 1949 Norwood procedures in 48 hospitals from the University HealthSystem Consortium (UHC) from 1998 to 2007. The hospital volume-outcome relationship was clearly supported by the findings for hospital mortality. Both low volume hospitals (OR 2.49, 95% CI 1.51-4.07) and medium volume hospitals (OR 1.75, 95% CI 1.23-2.49) had much higher mortality rates when compared with high volume hospitals (more than 30 procedures per year).

The same data source was used by Gutgesell & Gibson [35] who analyzed the period from 1990 to 1999. Their descriptive analysis of 40 hospitals showed a 10% point (50% vs. 40%) higher mortality rate in low volume hospitals (less than 50 procedures through study period) when compared to high volume hospitals.

The following decade (2000–2009) was analyzed with the STS-CHSDB [37,38]. Low and medium hospital volume revealed higher OR (low OR 1.37, medium OR 1.20) when compared to high volume hospitals, but both results were statistically not significant. Instead, a twofold decrease in hospital volume (treated as continuous variable) resulted in a significant finding for higher hospital mortality (OR 1.17, 95% CI 1.01-1.35). The analysis by Welke et al. [23] used the same data for a five-year period (2002 to 2006) and found statistically significant effects in favor of high volume hospitals. However, volume categories were not based on Norwood procedures but on pediatric cardiac surgeries.

Karamlou et al. [39] analyzed clinical data of 29 hospitals from 1994 to 2000. When treating hospital volume as a continuous variable an increase of one additional case per year showed no statistically significant effect on the mortality rate (p = 0.38).

The Pediatric Heart Network SVR trial showed inconclusive findings with respect to the hospital volume-outcome relationship [41]. On the one hand, low volume hospitals had a higher chance for patients suffering from sepsis or renal failure. On the other hand, medium and high volume hospitals had a lower chance when compared to very high volume hospitals. The result for renal failure only proved to be statistically significant for high volume hospitals vs. very high volume hospitals (OR 0.32, 95% CI 0.11-0.91). Linear regression models also showed inconsistent findings for the time to first extubation, the length of ventilation and the length of stay.

## Discussion

This article reviewed the existing literature on the volume-outcome relationship for the Norwood procedure, including specialization and regionalization. In general, these data demonstrate the presence of a volume-outcome relationship for the Norwood procedure. However, the magnitude of the volume effect is difficult to assess. It should also be kept in mind that volume is a proxy for quality and cannot fully explain differences between centers. Among other factors such as ownership, teaching status, location, size of the hospital, in particular center-specific effects might be able to explain more differences between centers [23,43,44]. The specialization and regionalization were studied less intensively. However, the results should be interpreted with caution for reasons outlined below.

### Hospital type

The influence of the hospital type was analyzed in two studies using the Kids Inpatient Database datasets 1997 and 2003. Both studies tend to support the hypothesis of better outcomes in teaching hospitals. The definition of teaching hospitals changed from 1997 to 2000. In the data of 2001, 20.1% of the hospitals were designated as teaching hospitals, as compared to 14.3% under the definition of 1997 [45]. Furthermore, new US states were added to the Kids Inpatient Database longitudinally, resulting in a higher percentage of US population covered in 2003 than in 1997. The impact of these differences on the findings is hard to assess. Nevertheless, the Kids Inpatient Database is known to be representative for the US [45], and thus, the study results show a tendency towards lower mortality rates in teaching hospitals, although it has to be acknowledged that the majority of Norwood procedures are performed in teaching hospitals.

### Hospital volume

All studies indicated a hospital volume-outcome relationship, most of them even having significant results. These data are meaningful, but each study has limitations. The single best study was done by Berry et al. [33] using the Kids Inpatient Database. They found a strong hospital volume-outcome relationship and their analysis was adjusted for a number of relevant risk factors.

### Surgeon volume

Surgeon volume was much less studied. Results are very heterogeneous. Only one study reported a significant result supporting the surgeon volume-outcome relationship for mortality when comparing the highest volume category with the lowest volume category [37,38]. The underlying STS-CHSDB is widely used for analyses in cardiac surgery [46]. We were not able to fully assess the quality of the database due to a lack of information concerning the validity and completeness. However, we are aware that the quality of the data source was described elsewhere [46-48] and found to be satisfactory.

It is important to notice that the study by Tabbutt et al. is a clinical trial with inconsistent findings across volume categories. However, the findings on the time to first extubation and the length of ventilation are in congruence with our hypothesis. Although the fourth study failed marginally to reach statistical significance for mortality, the absolute risk reduction (ARR) of 20% points is highly relevant [42]. Anyhow, this finding is difficult to interpret. On the one hand, the PHIS is a highly selective sample of more than 40 hospitals (29 hospitals at the time of writing of the study) throughout the US [49]. On the other hand, it includes the largest children’s hospitals in the US. Given that these hospitals are highly specialized, we would have expected a lower absolute risk reduction.

### Hospital or surgeon volume

This raises the question of whether surgeon volume might be more important than hospital volume. Multivariate models were applied in only two studies, one of them showing overall inconsistent findings with respect to our review question [41]. The second study found a statistically significant effect for surgeon volume, when adjusted for hospital volume, but not for hospital volume, when adjusted for surgeon volume [37,38].

### Strengths and weaknesses of the included studies

A number of issues that might have biased the study results should be kept in mind. First of all, taking volume as an outcome measure could be confusing as the number of performed procedures may classify the same hospital as low volume or high volume depending on the geographical area. This can make findings across studies difficult to compare.

Furthermore, surgeon and hospital volume were defined in several ways in our included studies. However, it has been shown that conclusions of hospital volume-outcome analyses are similar regardless of how hospital volume was defined [48]. There are no obvious reasons why this should differ with respect to surgeon volume. In addition, there is no specific ICD-9 or other procedure code that indicates a Norwood procedure, and therefore case definitions varied by study. However, it should be noted that the STS Congenital Heart surgery database does not rely on ICD-9 nor on other procedure codes. It contains very detailed data collection forms so that it can be concluded that the misclassification of the Norwood procedure is put to a limit compared with ICD-9 codes. Two studies used data from the STS-CHSDB [23,37].

It has been questioned whether administrative data is as good as clinical data to explore the volume-outcome relationship [47]. This is also why we assessed the quality of registry data. Nine out of eleven studies in our review were registry-based. The first clinical study had inconsistent findings [41], while the second used inappropriate statistics [39]. So, we are not able to judge whether this might have influenced our results.

### Limitations of the review

We acknowledge that our work has some limitations. First, we did not search for grey literature. This might have yielded additional information for other countries than the US, in particular. However, the Norwood procedure is rare due to their low incidence. Thus, meaningful analyses can be most expected from populous countries like the US. Nationwide samples from small countries would take much time to obtain a sufficient number of Norwood procedures for analysis. Changes in clinical practice would be likely to hamper the reliability of results. The low procedural volume is also reflected by the fact that even recently published studies are often based on much older data. In an outstanding case, the paper by Karamlou et al. [39] was published in 2010, being based on data from 1994 to 2000. The evidence presented in this systematic review is up-to-date, but our findings might not reflect the current clinical practice due to the time lag bias. Although this is a known issue in systematic reviews, the time lag bias is much more prominent in our systematic review. Furthermore, we can also not preclude the risk of overlapping databases. For instance, neonates included in the PHIS might also be included in the Kids Inpatient Database, introducing double counting of cases in our analysis (this does not affect our results for specialty centers and the hospital type).

We did not use a validated tool to appraise study quality but developed our own tool based on a previous Cochrane review, as there is no tool that can be considered the ‘gold standard’ for this kind of question. Assessing the quality of registry data was always assessed to have an unclear risk of bias due to limited information in the articles. We still believe this item to be of high relevance. As we described above, we found appropriate information in other sources than the included articles. It would require much effort to search for this information for each database. Information for older data sets might not even be available anymore. Thus, we believe it is the authors’ responsibility to provide appropriate information. However, it is not our intention to blame the authors for omitting this, as no reporting standard exists for registry-based studies.

## Conclusion

In conclusion, this systematic review supports the presence of a volume-outcome relationship in the Norwood procedure. However, the magnitude of the volume effect is difficult to assess. There are significant clinical effects with respect to mortality. The question whether hospital volume or surgeon volume is a better predictor for outcomes needs more investigation. A concentration of Norwood procedures could lead to a decrease in mortality, although there is no evidence for a specific volume cut-off. Since volume is nothing more than a proxy for quality of care, additional criteria should be taken into consideration in planning concentration initiatives.

### Ethics

The study did not involve any human subjects, human material, or human data.

## Competing interest

There is no conflict of interest to disclose.

## Authors’ contribution

DP conceptualized and designed the study, searched for literature, extracted and analyzed data, drafted the initial manuscript, and approved the final manuscript as submitted. TM searched for literature, extracted data and analyzed data, and approved the final manuscript as submitted. BA analyzed and interpreted data, critically reviewed the manuscript, and approved the final manuscript as submitted. All authors read and approved the final manuscript.

## Pre-publication history

The pre-publication history for this paper can be accessed here:

http://www.biomedcentral.com/1471-2431/14/198/prepub

## Supplementary Material

Additional file 1 Search strategy. Click here for file

Additional file 2 Critical appraisal tool. Click here for file

Additional file 3 List of excluded studies. Click here for file
