# Supplementary material for: A systematic review of the impact of volume of surgery and specialization in Norwood procedure
Source: BMC Pediatr. 2014 Aug 6;14:198. doi: 10.1186/1471-2431-14-198 (PMC4127072; doi:10.1186/1471-2431-14-198)
Supplement: Additional file 1 — Search strategy. [file 1471-2431-14-198-S1.doc]

**Appendix 1**

**Search strategy**

Embase

('Norwood procedure'/exp OR norwood:ab,ti OR 'hypoplastic left heart syndrome'/exp OR hlhs:ti,ab) AND (Volume:ti,ab OR size:ti,ab OR level:ti,ab OR type:ti,ab OR workload:ti,ab OR caseload:ti,ab OR centrali*:ti,ab OR decentral*:ti,ab OR regionali*:ti,ab OR speciali*:ti,ab) AND ('mortality'/exp OR 'survival'/exp OR 'postoperative complication'/exp OR 'treatment outcome'/exp OR mortality:ti,ab OR survival:ti,ab OR complication*:ti,ab OR outcom*:ti,ab)

Medline (via Pubmed)

("norwood procedures"[Mesh] OR Norwood[tiab] OR "Hypoplastic Left Heart Syndrome"[Mesh]/ surgery OR hlhs[tiab] OR “Hypoplastic Left Heart Syndrome”[tiab]) AND (Volume[tiab] OR size[tiab] OR level[tiab] OR type[tiab] OR workload[tiab] OR caseload[tiab] OR centrali*[tiab] OR decentral*[tiab] OR regionali*[tiab] OR speciali*[tiab]) AND ("Mortality"[Mesh] OR "Survival"[Mesh] OR "Disease-Free Survival"[Mesh] OR "Postoperative Complications"[Mesh] OR "Treatment Outcome"[Mesh] OR mortality[tiab] OR survival[tiab] OR complication*[tiab] OR outcom*[tiab] OR "Outcome and Process Assessment (Health Care)"[Mesh])

Cochrane library

(Norwood[tiab] OR Norwood Procedures[MeSH] OR MeSH descriptor Hypoplastic Left Heart Syndrome, this term only with qualifier: SU OR hlhs[tiab] OR Hypoplastic Left Heart Syndrome[tiab]) AND (Volume[tiab] OR size[tiab] OR level[tiab] OR type[tiab] OR workload[tiab]

OR caseload[tiab] OR centrali*[tiab] OR decentral*[tiab] OR regionali*[tiab] OR speciali*[tiab]) AND (MeSH descriptor Mortality explode all trees OR MeSH descriptor Survival explode all trees OR MeSH descriptor Postoperative Complications explode all trees OR MeSH descriptor Treatment Outcome explode all trees OR mortality[tiab] OR survival[tiab] OR complication*[tiab] OR outcom*[tiab] OR MeSH descriptor Outcome and Process Assessment (Health Care) explode all trees)
