# Supplementary material for: A systematic review of the impact of volume of surgery and specialization in Norwood procedure
Source: BMC Pediatr. 2014 Aug 6;14:198. doi: 10.1186/1471-2431-14-198 (PMC4127072; doi:10.1186/1471-2431-14-198)
Supplement: Additional file 2 — Critical appraisal tool. [file 1471-2431-14-198-S2.doc]

**Appendix 2: critical appraisal tool**

**Representativeness of study cohort**

The representativeness of participants in studies of colorectal/colon/rectal cancer patients were coded as:

Low risk: e.g. if study cohort was representative and not from selected groups

High risk: e.g. if somewhat representative or from selected groups

Unclear risk: e.g. if no description of cohort was reported

**Ascertainment of intervention**

The ascertainment of surgeon/hospital volume and surgeon specialization in included studies were coded as:

Low risk: e.g. if ascertainment of intervention was from study data or from structured interviews

High risk: e.g. if self reported

Unclear risk: e.g. if not reported

**Comparability of intervention and comparison**/**control group**

The comparability of participants in the intervention and comparison/ control groups were coded as:

Low risk: e.g. if study reported no differences between intervention and comparison/control group and/or if

adjustments for case-mix differences between intervention and comparison/control were performed

High risk e.g. if study reported significant differences between intervention and comparison/control group and/or if adjustments for case-mix differences between intervention and comparison/control were not performed

Unclear risk e.g. if differences between intervention and comparison/control groups were not reported

**Assessment of outcomes**

The assessments of primary and secondary outcomes in studies were coded as:

Low risk: e.g. if independent blind assessment of outcomes was performed or outcomes were assessed by the use of record linkage

High risk: e.g. if self reporting of outcomes was performed

Unclear risk: e.g. if no description was reported of how the outcomes were assessed.

**clinical studies:**

**Addressing incomplete data**

The proportion of participants whose outcomes were analysed in included studies were assessed and coded as:

Low risk: e.g. if loss of outcome data for participants or participants lost to follow-up was unlikely to introduce bias (<20%)

high risk: e.g. if loss of outcome data for participants or participants lost to follow-up was likely to introduce bias (>20%)

Unclear risk: e.g. if no information was provided

**Missing data on primary interventions and outcomes**

The proportion of participants in the intervention groups whose outcomes were analysed in included studies were assessed and coded as:

Low risk: e.g. if loss of data on primary interventions and outcomes was unlikely to introduce bias (<20%)

High risk: e.g. if loss of data on primary interventions and outcomes was likely to introduce bias (>20%)

Unclear risk: e.g. if no information was provided

**register based studies:**

**Quality of registry data**

The quality of registry data was coded as:

Low risk: e.g. if quality of data in terms of e.g. validation of data, missing values is described

High risk: e.g. if quality of data can be questioned

Unclear risk: e.g. if no information was provided

**Selection of patients**

The process how patients were selected from registry to be included in the study was coded:

Low risk: e.g. if selection of patients was clearly described and numbers illustrating the patient flow were given

High risk: e.g. if selection is described but lacks of clarity of plausibility

Unclear risk: e.g. if no information was provided
