# Supplementary material for: A systematic review of the impact of volume of surgery and specialization in Norwood procedure
Source: BMC Pediatr. 2014 Aug 6;14:198. doi: 10.1186/1471-2431-14-198 (PMC4127072; doi:10.1186/1471-2431-14-198)
Supplement: Additional file 3 — List of excluded studies. [file 1471-2431-14-198-S3.doc]

**Appendix 3**

**List of excluded studies**

*Reason for exclusion: study does not deal with the Norwood procedure*

Chang RKR, Rodriguez S, Lee M, Klitzner TS. Risk factors for deaths occurring within 30 days and 1 year after hospital discharge for cardiac surgery among pediatric patients. American heart journal. 2006;152(2):386-93.

MacDonald MJ, Reinhartz O, Hanley FL, Checchia PA. Effect of surgical case volume on outcome after the Norwood procedure [1] (multiple letters). Journal of Thoracic and Cardiovascular Surgery. 2005;130(6):1731-2.

Pasquali SK, Li JS, He X, Hodges A, Hornik CP, Jaquiss RDB, et al. Patient risk status, center volume and outcome following the Norwood operation. Journal of the American College of Cardiology. 2011;57(14):E406.

Scheurer M, Gauvreau K, Laussen PC, Mayer JE, Atz AM, Newburger JW. The impact of a designated cardiac intensive care unit on outcomes after the Norwood procedure. Journal of the American College of Cardiology. 2011;57(14):E405.

Tabbutt S, Ghanayem N, Cooper DS, Frank DU, Lu M, Frommelt P, et al. Risk factors for hospital mortality and morbidity following the norwood procedure: Results from the multicenter single ventricle reconstruction trial. Circulation. 2011;124(21).

*Reason for exclusion: study does not deal with volume, specialization or regionalization*

Connor JA, Arons RR, Figueroa M, Gebbie KM. Clinical outcomes and secondary diagnoses for infants born with hypoplastic left heart syndrome. Pediatrics. 2004 Aug;114(2):e160-5.

Ghanayem NS, Allen KR, Tabbutt S, Atz AM, Clabby ML, Cooper DS, et al. Interstage mortality after the Norwood procedure: Results of the multicenter Single Ventricle Reconstruction trial. J Thorac Cardiovasc Surg. 2012 Oct;144(4):896-906.

Pasquali SK, Ohye RG, Lu M, Kaltman J, Caldarone CA, Pizarro C, et al. Variation in perioperative care across centers for infants undergoing the Norwood procedure. Journal of Thoracic and Cardiovascular Surgery. 2012.
